# Specialized killing across the domains of life by the type VI secretion systems of *Pseudomonas aeruginosa*

**DOI:** 10.1042/BCJ20230240

**Published:** 2025-01-08

**Authors:** Jake Colautti, Steven D. Kelly, John C. Whitney

**Affiliations:** 1 Michael DeGroote Institute for Infectious Disease Research, McMaster University, Hamilton, ON, L8S 4K1, Canada; 2 Department of Biochemistry and Biomedical Sciences, McMaster University, Hamilton, ON, L8S 4K1, Canada; 3 David Braley Center for Antibiotic Discovery, McMaster University, Hamilton, ON, L8S 4K1, Canada

**Keywords:** Pseudomonas aeruginosa, Type VI secretion systems, Bacterial toxins

## Abstract

Type VI secretion systems (T6SSs) are widespread bacterial protein secretion machines that inject toxic effector proteins into nearby cells, thus facilitating both bacterial competition and virulence. *Pseudomonas aeruginosa* encodes three evolutionarily distinct T6SSs that each export a unique repertoire of effectors. Owing to its genetic tractability, *P. aeruginosa* has served as a model organism for molecular studies of the T6SS. However, *P. aeruginosa* is also an opportunistic pathogen and ubiquitous environmental organism that thrives in a wide range of habitats. Consequently, studies of its T6SSs have provided insight into the role these systems play in the diverse lifestyles of this species. In this review, we discuss recent advances in understanding the regulation and toxin repertoire of each of the three *P. aeruginosa* T6SSs. We argue that these T6SSs serve distinct physiological functions; whereas one system is a dedicated defensive weapon for interbacterial antagonism, the other two T6SSs appear to function primarily during infection. We find support for this model in examining the signalling pathways that control the expression of each T6SS and co-ordinate the activity of these systems with other *P. aeruginosa* behaviours. Furthermore, we discuss the effector repertoires of each T6SS and connect the mechanisms by which these effectors kill target cells to the ecological conditions under which their respective systems are activated. Understanding the T6SSs of *P. aeruginosa* in the context of this organism’s diverse lifestyles will provide insight into the physiological roles these secretion systems play in this remarkably adaptable bacterium.

## Introduction

Bacteria live in complex environments within which they must compete for limited resources, establish and defend ecological niches and perturb the physiology of host cells during infection. To accomplish these tasks, some bacteria secrete toxic proteins using several sophisticated protein secretion systems that have evolved for this purpose [1–3]. The type VI secretion system (T6SS) is one such apparatus that is widespread in Gram-negative bacteria and functions to deliver a cocktail of toxic proteins known as effectors directly into adjacent cells [4,5]. By targeting conserved essential processes, including cell wall homeostasis, protein translation, and oxidative ATP synthesis, these effectors kill a broad range of target cells and, thus, contribute to microbial competition and virulence [6–8].

**Figure 1:**
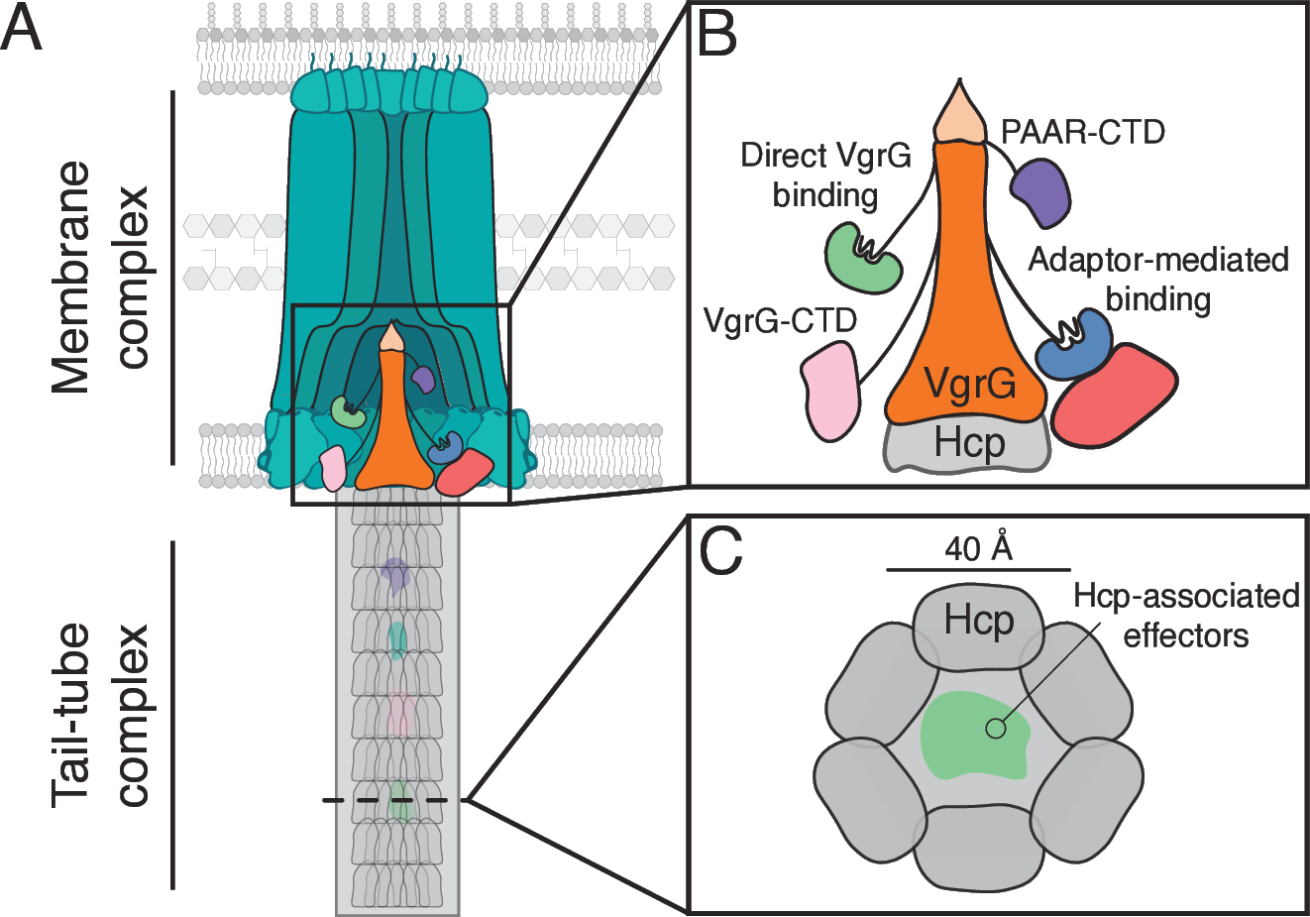
The structure of the T6SS. (**A**) Schematic representation of the general T6SS membrane complex and tail tube complex. The baseplate complex that connects these two sub-assemblies has been omitted for clarity. (**B**) Schematic representation of characterized mechanisms of effector recruitment to the spike complex. While some effectors exist as C-terminal domains (CTD) of proline-alanine-alanine-arginine (PAAR) or valine glycine repeat protein G (VgrG) proteins, others are recruited to the complex by direct interaction or via interactions with an adaptor protein. (**C**) Cross-sectional representation of the haemolysin co-regulated protein (Hcp) tube containing Hcp-associated effectors.

Owing to its genetic tractability and facile growth requirements, *Pseudomonas aeruginosa* has emerged as a key model organism for studies of T6SS function. Furthermore, *P. aeruginosa* encodes three genetically distinct T6SSs, known as the H1-, H2-, and H3-T6SS, that each export a unique repertoire of effectors [9,10]. These systems have provided a wealth of opportunities to study the general T6SS structure and function at a molecular level. Research using this organism has rapidly advanced our understanding of how T6SSs recruit and export effectors and how these effectors kill target cells (Figure 1). In addition to being a tractable model organism, *P. aeruginosa* is also an important environmental and pathogenic bacterium that inhabits a broad range of ecosystems and tolerates numerous environmental stressors [11–13]. The study of the *P. aeruginosa* T6SSs has, therefore, shed light on the roles these systems play in the diverse lifestyles of this organism. Here, we review the current knowledge of these three T6SSs and present evidence that each system serves a distinct physiological function in *P. aeruginosa*. The collective evidence supports the H1-T6SS as a dedicated weapon of interbacterial competition that is most active in free-living cells whereas the less well-characterized H2- and H3-T6SSs appear to function primarily during *P. aeruginosa* infection of mammals. In support of this model, we first discuss the signals that regulate each T6SS and explore the co-ordination of these systems with other *P. aeruginosa* behaviours including biofilm formation, nutrient acquisition, and virulence. Second, we compare the effector repertoires of each T6SS and discuss the range of organisms that can potentially be targeted by these distinct repertoires. Finally, we explore several outstanding questions regarding the T6SSs of *P. aeruginosa* and propose future directions for research in this field.

## The structure and the function of the T6SS

In recent years, extensive research on the T6SSs harboured by *P. aeruginosa* and other model organisms has shed light on the structure of this system and the mechanisms by which effectors are delivered between bacteria (Figure 1). The T6SS comprises two macromolecular assemblies: a membrane-bound apparatus and a tail tube complex that is injected into the target cell along with its associated effectors. The membrane apparatus is composed of the inner membrane proteins TssL and TssM and the outer membrane lipoprotein TssJ, which together span the cell envelope and enable the delivery of effectors from the cytoplasm of the producing cell directly into the target cell, bypassing the producing cell periplasm [14]. The cytoplasmic surface of TssL interacts with the baseplate complex, which serves as a nucleation point for the polymerization of the tail tube complex and its surrounding sheath [15]. The tail tube complex is a hollow cylinder of stacked rings of hexameric haemolysin co-regulated protein (Hcp) capped by a trimer of valine glycine repeat protein G (VgrG) [16]. This trimer is ‘sharpened’ by a single copy of the zinc-binding proline-alanine-alanine-arginine (PAAR) protein, which likely facilitates penetration of the target cell membrane [17]. The tail tube complex is surrounded by a sheath comprised of the proteins TssB and TssC and the contraction of this sheath expels the tail tube complex and its associated effectors through the membrane apparatus into the target cell [18]. Many of the molecular events underlying these processes have been thoroughly characterized and have been meticulously reviewed elsewhere [4,19].

Several mechanisms of effector recruitment to the tail tube complex have been described. The best characterized is Hcp, which serves as a secreted chaperone that accommodates small effectors within its ~4 nm lumen, thus delivering these effectors into the target cell [20]. However, effectors that transit the T6SS by this mechanism cannot be larger than the diameter of the Hcp ring. Consequently, larger effectors instead associate with the outer surface of the tail tube complex for secretion. Some effectors exist as C-terminal domains of VgrG or PAAR proteins and are, therefore, evolutionarily fused to these tail tube proteins [16,21]. Others, however, are encoded by distinct genes and, therefore, must interact with the tail tube complex through non-covalent interactions. In some instances, these interactions require the activity of dedicated trafficking domains or accessory proteins, such as those belonging to the T6SS adaptor protein (Tap) or DUF2345 families [22–29]. Tap proteins contain a structurally conserved N-terminal lobe that binds to a short extension protruding from VgrG or PAAR proteins, and a variable C-terminal lobe that has evolved to recognize structurally distinct effectors [23,26,29]. Although there is some evidence suggesting that DUF2345 domains similarly enable the recruitment of effectors to their cognate VgrG proteins, the precise molecular contacts underlying these interactions remain unknown.

In general, the mechanisms by which T6SS effectors are recognized and exported are well described but much less is known about how effectors localize to the appropriate cellular compartment following delivery. Effectors that act in the target cell periplasm do not appear to contain additional domains that enable outer membrane translocation, suggesting that these effectors are delivered directly into the periplasm by the T6SS apparatus [6,30]. By contrast, effectors that act intracellularly often require additional factors for entry into the target cell cytoplasm. Some such effectors contain transmembrane domains that are proposed to be inserted into the inner membrane following T6SS-mediated delivery into the target cell periplasm [31,32]. These transmembrane domains are thought to enable translocation of the effector’s toxin domain into the cytoplasm [32]. Additionally, several proteins belonging to the recombination hotspot (Rhs) family of proteins function as cytoplasmic T6SS effectors [33–35]. These effectors are defined by the presence of a large β-cage domain that encapsulates a toxin domain prior to delivery to the recipient cell [36]. In several characterized examples, this cage domain has been proposed to facilitate correct subcellular localization of the toxin domain following T6SS delivery [37,38]. Besides the above examples, additional cytoplasmic effectors that do not contain such domains have been described [39]. Therefore, it remains unclear how these effectors access the target cell cytoplasm following T6SS delivery.

## Regulation of the *P. aeruginosa* T6SSs

**Figure 2:**
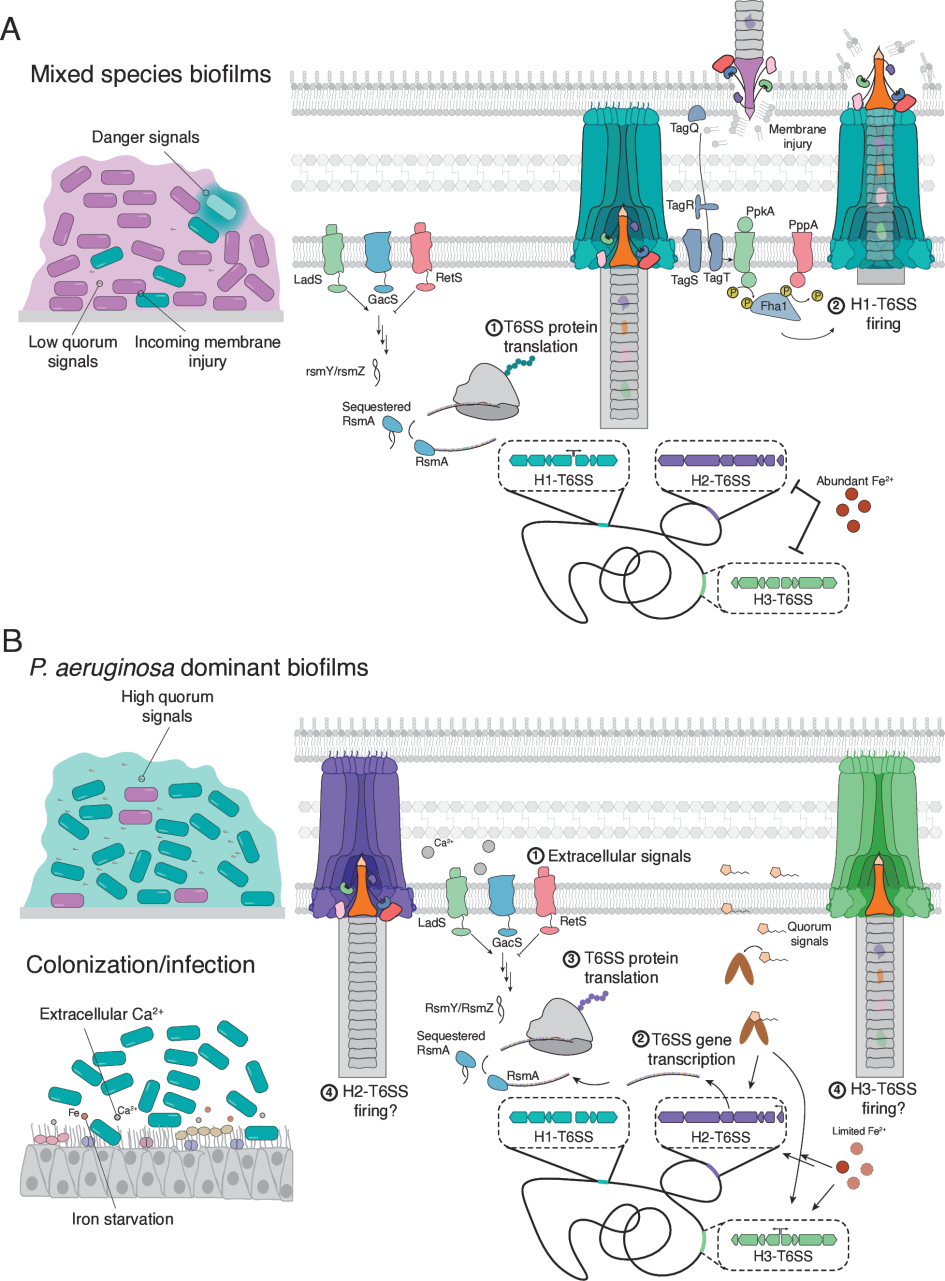
Regulatory inputs that control T6SS activity under different environmental conditions. (**A**) In conditions where *P. aeruginosa* is not a dominant member of the microbial community, the H1-T6SS serves as a weapon of defensce against incoming threats. The absence of quorum signals induces transcription of H1-T6SS genes, whereas the relative abundance of iron inhibits transcription of the H2- and H3-T6SS loci. Signalling through the Gac/Rsm pathway induces the expression of *rsmY* and *rsmZ*, which sequester the RNA-binding protein RsmA and enable the translation of T6SS proteins. Finally, signalling through the threonine phosphorylation pathway co-ordinates H1-T6SS firing with incoming membrane injuries, thus enabling *P. aeruginosa* to engage in tit-for-tat T6SS behaviour. (**B**) Under conditions where *P. aeruginosa* dominates the microbial community, the H2- and H3-T6SSs serve as weapons of co-ordinated attack against non-kin cells or an animal host. Extracellular signals of high *P. aeruginosa* density and the host environment, such as iron starvation, quorum signals, and extracellular calcium, induce transcription and translation of H2- and H3-T6SS genes via the Gac/Rsm and Fur pathways.

Although some clinical *P. aeruginosa* isolates constitutively export Hcp, laboratory strains typically do not harbour active T6SSs under standard growth conditions [10,40]. Therefore, the mechanistic study of these systems has relied on strains lacking repressors of T6SS gene expression to induce T6SS activity under laboratory conditions. The characterization of these regulatory genes has identified several signalling cascades that act at the transcriptional, translational and post-translational levels to control T6SS gene expression and effector export (Figure 2) [40–44]. Although these signalling pathways have been well characterized at the molecular level, only recently have some of the environmental cues that they sense and respond to been identified [42,45,46]. Therefore, the physiological role of each T6SS has been challenging to elucidate because the natural conditions that induce each system remain elusive. However, T6SS activity is co-ordinated with that of other systems for which physiological functions are better understood, including systems involved in biofilm formation, nutrient sensing and virulence. Understanding the global regulatory programs that co-ordinate the expression of these genes with that of T6SS genes can, therefore, provide insight into the possible ecological roles of each T6SS. To this end, we have examined the regulatory inputs that control T6SS activity and framed them in the context of other co-regulated genes and behaviours in *P. aeruginosa*.

### Gac/Rsm signalling

Current evidence suggests that *P. aeruginosa* typically adopts one of two major lifestyles: a planktonic lifestyle characterized by the rapid growth of isolated cells in liquid conditions or a sessile lifestyle in which cells grow as an aggregate enclosed within a macromolecular matrix known as a biofilm [47]. These distinct lifestyles allow *P. aeruginosa* to thrive in both aquatic and terrestrial environments. Furthermore, in a mammalian host, these lifestyles are associated with diametric pathogenic strategies; planktonic cells can cause severe, acute *P. aeruginosa* infection whereas sessile growth supports the maintenance of chronic disease. This transition is co-ordinated by a global regulatory system known as Gac/Rsm [48]. Briefly, planktonic growth is maintained by the RNA-binding protein, RsmA, which binds to and translationally silences mRNAs involved in biofilm formation [49]. Transition to the sessile lifestyle is driven by the activation of the GacS/GacA two-component system, leading to transcription of the regulatory RNAs *rsmY* and *rsmZ* [50]. These RNA molecules bind to and sequester RsmA away from its target mRNAs, thus permitting translation of the encoded proteins [51]. This pathway is fine-tuned by two additional protein sensors in the inner membrane, LadS and RetS, which stimulate and inhibit GacS signalling, respectively [41,48,50,52]. The presence of multiple membrane-bound sensors enables the Gac/Rsm pathway to integrate inputs from multiple extracellular signals and co-ordinate *P. aeruginosa* gene expression accordingly. The activation of the Gac/Rsm pathway induces expression of genes required for the formation of *P. aeruginosa* biofilms and represses acute virulence factors and motility systems involved in the planktonic lifestyle, such as the type III secretion system, flagellum and type IV pilus [41,52]. Consistent with this function in the transition from acute to chronic infection, the Gac/Rsm system has been shown to respond to environmental cues found in the host environment, such as hypoxia, calcium and mucin glycans [45,46,53].

T6SS-mediated protein delivery requires direct contact between the producing and recipient cells [39]. It is, therefore, not surprising that growth in a biofilm, where cell-to-cell contact is abundant, induces gene expression of all three T6SSs in a Gac/Rsm-dependent manner [40,41]. The deletion of several inhibitory genes in the Gac/Rsm pathway, such as *retS* and *rsmA*, derepresses the signalling pathway, increasing transcription and translation of the H1-, H2- and H3-T6SSs and enhancing T6SS-dependent competitive fitness against susceptible bacteria [40]. While Gac/Rsm signalling is directly implicated in the translational regulation of T6SS proteins, the observation that this pathway also modulates levels of T6SS mRNA implicates additional transcriptional regulators in the control of this system. Indeed, in the case of the H1-T6SS, RsmA represses the translation of *amrZ* transcripts, thereby reducing the cellular abundance of the H1-T6SS positive regulator, AmrZ [49]. Thus, Gac/Rsm signalling controls T6SS expression using a two-tiered mechanism involving direct control of T6SS protein translation as well as indirect control of T6SS gene transcription.

The co-regulation of the T6SS and factors involved in biofilm production suggest an important role for the T6SS in the sessile growth behaviour of *P. aeruginosa*. The sensing of and response to potential danger in a densely populated biofilm community is one proposed role for these systems in this context [43]. By delivering antibacterial effectors to adjacent cells, *P. aeruginosa* can eliminate non-kin cells that pose a threat to the biofilm community. Consistent with this idea, H1-T6SS gene expression was found to be stimulated by co-culture with *Burkholderia thailandensis* strains that also harbour a T6SS [43]. It was subsequently shown that effectors delivered to *P. aeruginosa* by the *B. thailandensis* T6SS lyse a subset of the *P. aeruginosa* population, which release signals of cell injury that induce H1-T6SS expression in surviving cells [43]. This phenomenon requires signalling through the Gac/Rsm pathway, consistent with the H1-T6SS being used as a response to perceived danger in a biofilm environment. *P. aeruginosa* lysate has also been shown to induce phage defence systems via Gac/Rsm signalling, which further implicates Gac/Rsm as a sensor of danger signals [54]. Together, these observations suggest that the Gac/Rsm pathway induces a stress response to signals of danger that includes systems involved in defence against incoming bacterial and viral threats. The finding that *P. aeruginosa* lysate induces H1- but not H2- or H3-T6SS activity suggests that these latter systems are likely not a component of this defensive response and implies that they serve a different function in a sessile lifestyle. Further investigation is required to better define the mechanisms by which the Gac/Rsm signalling is fine-tuned to selectively induce H1-T6SS activity upon exposure to kin cell-derived danger signals.

### Quorum sensing

Discriminating between kin and non-kin cells is a critical component of microbial life [55]. Consequently, bacteria have evolved sophisticated systems that allow them to sense the density and composition of the microbial community they inhabit. These so-called quorum sensing systems consist of a secreted autoinducer molecule, the concentration of which correlates with cell density, and a response regulator protein [56,57]. Once the concentration of autoinducer reaches a critical threshold, the autoinducer binds to and activates the response regulator, thus inducing the expression of quorum-regulated genes. In this way, a population of bacteria can initiate collective behaviours upon reaching an appropriate cell density.

Quorum sensing controls the expression of several important *P. aeruginosa* virulence factors [58,59]. These include proteins involved in the establishment and maintenance of *P. aeruginosa* infection, such as elastases, phospholipases and exotoxins, as well as secondary metabolites that enable survival in the host (reviewed by Moradali et al. [59]). The finding that these virulence factors are all regulated by quorum sensing pathways implies that *P. aeruginosa* virulence is a collective attack mounted against a host by a bacterial population upon reaching a critical density. Consistent with this idea, disruption of quorum sensing by genetic manipulation or treatment with small molecule quorum sensing inhibitors attenuates *P. aeruginosa* virulence [60,61].

Like the Gac/Rsm pathway, quorum sensing has been shown to control all three T6SSs in *P. aeruginosa* [42,62]. However, unlike Gac/Rsm, which induces expression of all three systems, quorum signals differentially regulate the *P. aeruginosa* T6SSs. The H1-T6SS is inhibited by quorum sensing molecules whereas the H2- and H3-T6SSs are induced by these signals [42,62]. The finding that the H1-T6SS is inhibited by quorum sensing suggests that this system is likely not involved in collective behaviours but rather functions at low *P. aeruginosa* density such as during the formation of a new biofilm or in microbial communities where *P. aeruginosa* is not the dominant species. By contrast, the finding that the H2- and H3-T6SSs are induced by quorum signalling suggests that these systems may be components of the larger virulence strategy that enables a *P. aeruginosa* population to establish infection. However, as we discuss below, future investigation will be required to uncouple the direct host-targeting effect of the H2- and H3-T6SSs from their potential role in outcompeting the commensal microbiome.

### Iron availability

Iron is an essential nutrient required by most bacteria for survival. Therefore, bacteria have evolved systems that sense and sequester environmental iron when it is limited [63]. To prevent bacterial growth and virulence, eukaryotic organisms have evolved mechanisms to limit bacterial access to iron in a process known as nutritional immunity [63]. Iron is a limited nutrient in the host environment and low iron availability serves as a signal that induces virulence behaviours in pathogenic organisms including *P. aeruginosa*.

Conflicting evidence exists regarding the role of iron in the regulation of the *P. aeruginosa* T6SSs [42,64,65]. Growth in iron-rich conditions has been shown to inhibit the translation of proteins belonging to all three T6SSs, although this effect appears to be most profound for the H2-T6SS, which is induced by iron starvation [42,65]. The induction of the H2-T6SS under conditions of iron starvation supports a role for this system in virulence because iron limitation is a signal of the host environment that induces multiple *P. aeruginosa* virulence factors [66,67]. However, other researchers have reported that iron excess, rather than limitation, induces H2-T6SS gene expression [64]. These investigators found that iron released upon epithelial cell injury during viral infection specifically induces expression of the H2-T6SS effector TseT [64]. The induction of this T6SS effector may allow *P. aeruginosa* to outcompete nearby bacteria upon injury to its mammalian host and, thus, enable survival in this hostile environment during periods of host stress [64]. Further investigation is therefore required to understand the role of iron in regulating the H2-T6SS. Additionally, little is known about the role of iron in the regulation of the H1- and H3-T6SSs, so future research focusing on understanding how iron availability or limitation influences the activity of these systems may reveal new insights into their regulation.

### Tit-for-tat

The co-ordinated activity of the signalling pathways described above allows *P. aeruginosa* to tightly control the synthesis of T6SS proteins, thus ensuring that these systems are only assembled under conditions in which they are likely to prove useful. However, these regulatory pathways are unable to precisely control the timing of T6SS firing following assembly. While the exact mechanisms controlling the firing of the H2- and H3-T6SSs remain unknown, a sophisticated system that controls the firing of an assembled H1-T6SS apparatus has been described. This pathway, known as the threonine phosphorylation pathway (TPP), enables a ‘tit-for-tat’ behaviour in *P. aeruginosa* in which an assembled H1-T6SS will fire only in response to cell envelope injury [44,68].

The TPP consists of the membrane-bound kinase, PpkA, and its cognate phosphatase, PppA, which together regulate phosphorylation of the cytoplasmic protein Fha1 [44]. Upon sensing cell envelope injury, PpkA dimerizes and autophosphorylates before subsequently phosphorylating Fha1. Phosphorylated Fha1 localizes to the assembled H1-T6SS apparatus and triggers contraction of the T6SS sheath by an unknown molecular mechanism [44]. Thus, PpkA transduces signals from the cell membrane to the H1-T6SS apparatus and triggers the firing of the assembled apparatus. PppA directly counteracts the activity of PpkA by dephosphorylating Fha1, which inhibits H1-T6SS firing.

Several proteins present in the periplasm and both the inner and outer membranes modulate the activity of PpkA and, thus, control the firing of an assembled H1-T6SS. These proteins, referred to as TagQ, R, S and T, are thought to sense molecular events in the cell envelope and trigger H1-T6SS firing by activation of PpkA [69–71]. Consistent with this role, several cell envelope stressors have been shown to stimulate H1-T6SS firing through this pathway including incoming T6SS attacks [68], cell surface contact by conjugative pili [72] and perturbation of membrane homeostasis [73,74]. Although the precise molecular mechanisms by which TPP components sense and respond to these stressors remain unknown, these findings suggest that H1-T6SS firing is tightly controlled to ensure this system is activated only in response to an imminent threat. Together with the aforementioned finding that H1-T6SS gene expression is induced by danger signals released upon kin cell lysis, these data strongly suggest that the H1-T6SS is used to defend against microbial threats.

While post-translational control of the H1-T6SS has been extensively studied, less is known about if or how H2- and H3-T6SS firing is post-translationally regulated. The H2-T6SS gene cluster encodes a homolog of *fha1*, known as *fha2*, but appears to lack homologs of *ppkA* or *pppA* [44]. This observation suggests that the H2-T6SS is likely not controlled by a TPP since the H2 gene cluster does not encode the minimal components of such a system [44]. Consistent with this idea, outer membrane injury induced by magnesium chelation stimulates the H1- but not the H2- or H3-T6SSs [74]. This finding suggests that the H2- and H3-T6SSs may act as offensive weapons that strike a target cell first rather than defensive weapons like the H1-T6SS. Further investigation is required to conclusively demonstrate that this is the case for the H2- and H3-T6SSs and to identify other factors that may influence post-translational control of these systems.

In summary, several regulatory inputs control the activity of the T6SSs in *P. aeruginosa*. All three systems appear to constitute part of a sessile lifestyle, likely because T6SS protein delivery requires direct contact between the producing and recipient cells. H1-T6SS expression is induced by low *P. aeruginosa* cell density and external danger signals, and the assembled apparatus fires in response to incoming cell envelope attacks. These observations suggest that this system serves to defend *P. aeruginosa* against external microbial threats. By contrast, the H2- and H3-T6SSs appear to constitute part of a virulence regulatory program that allows a community of *P. aeruginosa* to mount a co-ordinated offensive attack against microbial competitors or a host organism.

## The T6SSs of *P. aeruginosa* export functionally distinct toxin repertoires

In addition to understanding the regulatory pathways that control the three T6SSs of *P. aeruginosa*, the biochemical activities of the effectors exported by these systems have been the focus of intense study in recent years. Interestingly, each T6SS exports effectors that target different cellular processes and act by distinct biochemical mechanisms. Considering the differential regulation of each system, this divergence in effector function is probably reflective of the physiological roles of each T6SS. In the following sections of this review, we explore the toxin repertoire of each T6SS and connect the target cell types and biochemical activities of the exported toxins to the biological contexts in which these systems are activated. The molecular mechanisms underlying toxin function will not be discussed in explicit detail as they have been aptly reviewed elsewhere [1,75].

### The H1-T6SS: a dedicated weapon for interbacterial competition

The regulation of H1-T6SS activity implicates it as a dedicated weapon of interbacterial competition that allows *P. aeruginosa* to inhibit the growth of competitor bacteria. Consistent with this proposed role, the H1-T6SS has been shown to export seven effectors that all contribute to the competitive fitness of * P. aeruginosa* against susceptible organisms (Table 1).

**Table 1:** Effector repertoires of the H1-, H2- and H3-T6SSs.

T6SS	Secretion mechanism	PAO1 locus tag	PA14 locus tag	Effector delivered	Citation
H1-T6SS	VgrG1a	PA0093	PA14_43090	Tse6 (PAO1) Tas1 (PA14)	[21,76,77]
	VgrG1b	PA0095	PA14_01160	Tse7	[78]
	VgrG1c	PA2685	PA14_29390	Tse5	[21,37,79]
	Hcp1	PA1844	PA14_40660	Tse1	[6,39]
	Hcp1	PA2702	PA14_29200	Tse2	[39]
	Hcp1	PA3484	PA14_19020	Tse3	[6,39]
	Hcp1	PA2774	PA14_28210	Tse4	[21,80]
H2	VgrG2a	PA1511	PA14_44900	Tle4/TplE	[81]
	VgrG2b	PA0262	PA14_03220	Tle3, VgrG2b toxin	[82,83]
	VgrG4a	PA3294	PA14_21450	Tle1	[30]
	VgrG4b	PA3486	PA14_18985	Tle5a/PldA	[30,84]
	VgrG5	PA5090	PA14_67220	Tle5b/PldB	[84]
	VgrG6	PA5265	PA14_69520	Ptx2	[29]
	VgrG14		PA14_43080	RhsP2	[35,85]
H3	VgrG3	PA2373	PA14_33960	TseF, TepB	[86,87]

The first T6SS effectors to be discovered and characterized were the H1-T6SS effectors (Tse) 1, Tse2 and Tse3 [39]. Tse1 and Tse3 lyse target bacteria by degrading peptidoglycan, a nearly universally conserved component of the bacterial cell wall that protects against osmotic stress and maintains cell shape [6,88]. While these effectors allow the H1-T6SS to target bacterial cells, they are ineffective against eukaryotic organisms since peptidoglycan is uniquely present in bacteria. Unlike Tse1 and Tse3, Tse2 does not target a molecule unique to bacteria [39]. Although its precise molecular mechanism of growth inhibition remains unknown, heterologous expression of Tse2 inhibits the growth of bacterial, fungal and mammalian cells, which indicates that this toxin targets a substrate that is conserved between these domains of life [39]. However, the H1-T6SS does not appear to deliver Tse2 to mammalian or yeast cells during co-culture with *P. aeruginosa* [39]. This finding suggests that, although Tse2 acts on a substrate present in eukaryotic cells, it is probably not delivered to these cells by the H1-T6SS. This finding further supports the model that the H1-T6SS strictly contributes to interbacterial competition and does not directly participate in *P. aeruginosa* virulence.

Since the initial characterization of Tse1-3, the mechanisms by which other H1-T6SS effectors inhibit target cell growth have been elucidated. Tse4 and Tse5 both depolarize the cytoplasmic membrane of target bacteria, thus dissipating the proton motive force and uncoupling oxidative electron transport from ATP synthesis [34,80]. Tse6 disrupts central metabolism by rapidly hydrolysing the electron carriers NAD(P)^+^ [76]. Lastly, Tse7 degrades chromosomal DNA upon delivery to target bacteria, although the precise mechanism of its nuclease activity remains poorly understood [78]. Interestingly, a subset of *P. aeruginosa* strains related to the laboratory strain PA14 encode the effector Tas1 instead of Tse6 [77]. This effector uses the essential nucleotides ADP and ATP to synthesize adenosine tetra- and pentaphosphate (ppApp and pppApp, respectively), which inhibits target cell growth by rapidly depleting energy stores [77]. The presence of this effector in a subset of *P. aeruginosa* isolates indicates that the H1-T6SS can export a more diverse effector repertoire than can be appreciated by strictly studying laboratory strains of this organism [89]. This uncharacterized pool of effectors represents an exciting area for future study because they may harbour enzymatic activities never before observed in nature or target previously overlooked physiologic processes in bacteria that could be exploited for future antimicrobial development.

### The H2-T6SS: a protein secretion system that targets multiple domains of life

In contrast to the H1-T6SS, the H2-T6SS delivers effectors to both bacterial and eukaryotic cells, thus contributing to virulence as well as interbacterial competition. This property is consistent with the finding that the H2-T6SS is induced by signals of the host environment together with many other virulence genes. Furthermore, *P. aeruginosa* strains lacking a functional H2-T6SS display impaired virulence in several infection models, including *Caenorhabditis elegans*, *Arabidopsis thaliana*, and mammalian lung and soft tissue models [62,90]. Importantly, this role in virulence is maintained in tissue culture systems that do not include a commensal lung microbiome, indicating that the H2-T6SS participates in the infection process by directly targeting host cells [90]. Taken together with the finding that the H2-T6SS also delivers effectors to adjacent bacteria, these data strongly implicate the H2-T6SS as a secretion system that targets cells from multiple domains of life [30].

Five of the eight H2-T6SS effectors characterized to date degrade membrane phospholipids [30,81,84,91,92]. These lipase effectors act by diverse biochemical mechanisms and together constitute a cocktail of enzymes that degrade phospholipids at multiple chemical linkages, leading to disruption of the target cell membrane [30]. Like the effectors exported by the H1-T6SS, these lipases confer a fitness advantage on *P. aeruginosa* in co-culture with susceptible competitor bacteria [30,81,84,91,92]. However, since many phospholipids are conserved between bacteria and eukaryotes, it is perhaps unsurprising that lipase effectors can also directly kill eukaryotic cells [81,92]. The finding that lipases are abundant among the H2-T6SS repertoire but absent among the known effector repertoires of the other *P. aeruginosa* T6SSs suggests that the H2-T6SS may have evolved to target both bacterial and eukaryotic cells.

While it is well established that lipases exported by the H2-T6SS contribute to *P. aeruginosa* virulence, their precise role in this complex process remains incompletely understood [81,92,93]. Upon delivery to mammalian epithelial cells by the H2-T6SS, Tle4 causes cell death by degrading phospholipids in the endoplasmic reticulum [81]. While the lethal effect of a phospholipase on epithelial cells might be expected, the finding that the H2-T6SS can deliver Tle4 directly into these cells suggests that they may represent a physiologically relevant target of this system. The H2-T6SS has also been found to deliver the lipases Tle5a and Tle5b to mammalian epithelial cells. Unlike Tle4, however, Tle5a and Tle5b do not kill the target cell but instead were shown to activate a signalling cascade that promotes *P. aeruginosa* internalization into the epithelium, an effect that was independent of lipase activity [92]. This is a curious finding, as it implies that these effectors have evolved two distinct activities – phospholipase activity that specifically kills bacterial competitors and a yet unknown biochemical activity that selectively manipulates the physiology of a eukaryotic host. Understanding the molecular basis for these seemingly disparate biochemical activities represents an exciting area for future research, as it may provide insight into a previously overlooked bacterial pathogenesis strategy.

Beyond its broad repertoire of phospholipases, the H2-T6SS exports four additional effectors: TseT, which is predicted to act as a deoxyribonuclease, the metalloprotease VgrG2b, the ADP-ribosyltransferase RhsP2, and the toxin of unknown function Ptx2 [23,82,85,29]. The C-terminal zinc metalloprotease toxin domain of VgrG2b disrupts bacterial morphology and cell division when expressed in the periplasm. However, the precise molecular target of VgrG2b remains elusive. Available evidence suggests that this toxin does not target peptide cross-links in the bacterial cell wall but may instead target inner membrane lipoproteins involved in bacterial cell division [82]. However, further investigation is required to better understand the molecular mechanism by which VgrG2b kills target bacteria. VgrG2b has also been found to perturb the epithelial cell cytoskeleton, thus promoting *P. aeruginosa* internalization into these cells [94]. While this activity has been attributed to the C-terminal metalloprotease domain of VgrG2b, the X-ray crystal structure of this domain does not reveal structural features beyond its protease domain that would enable its interaction with the mammalian cytoskeleton [82]. Therefore, like Tle5a/b, VgrG2b represents a unique opportunity to study mechanisms by which a single protein differentially perturbs bacterial and eukaryotic cells.

RhsP2 is the most recently characterized H2-T6SS toxin. This toxin adds ADP-ribose moieties to structured non-coding RNA molecules, such as tRNA, rRNA and the mRNA-processing ribozyme ribonuclease P [85]. By covalently modifying these essential molecules with bulky ADP-ribose groups, RhsP2 disrupts translation and mRNA processing, thereby inhibiting target cell growth. RhsP2 is unique among known RNA-targeting ADP-ribosyltransferase toxins in that it is highly promiscuous, modifying a wide range of cellular targets [7,85]. Further research is required to determine the sequence motif(s) that RhsP2 recognizes to better understand the mechanism underlying this promiscuity. However, consistent with its activity as a broadly acting RNA-modifying enzyme that disrupts several conserved cellular processes, the heterologous expression of RhsP2 is toxic to both bacterial and eukaryotic cells [85,95]. This finding is consistent with a possible role for RhsP2 as an effector that targets both bacterial and eukaryotic cells, although it remains to be shown whether RhsP2 is delivered to eukaryotic cells via the H2-T6SS.

The apparent role of the H2-T6SS in effector delivery to bacterial and eukaryotic cells represents an interesting biological phenomenon and a unique opportunity for further study. There is certainly no question that the H2-T6SS delivers effectors to bacteria and contributes to interbacterial competition. All H2-T6SS effectors characterized to date are encoded adjacent to cognate immunity proteins that confer resistance to their activity, which indicates that *P. aeruginosa* must protect itself against these effectors. Additionally, all well-characterized H2-T6SS effectors confer a fitness advantage on *P. aeruginosa* against competitor bacteria, which is consistent with the general role of the T6SS in interbacterial competition. However, accumulating evidence implicates the H2-T6SS in virulence as well as interbacterial antagonism, and the finding that the effector repertoire of this system is dominated by lipases, which are absent among the H1- and H3-T6SS effector repertoires, further supports the idea that the H2-T6SS constitutes a trans-kingdom targeting secretion system. The finding that H2-T6SS is induced by signals of the host environment in concert with other virulence genes further solidifies the role of this system in *P. aeruginosa* virulence. A better understanding of the molecular mechanisms by which H2-T6SS effectors kill or perturb host cells will provide deeper insight into the role these proteins play in *P. aeruginosa* virulence and may shed new light on the overall mechanism of *P. aeruginosa* pathogenesis.

### The H3-T6SS: a final frontier for type VI secretion research in * P. aeruginosa*?

While the toxin repertoires and potential biological functions of the H1- and H2-T6SSs have been well studied, much less is known about the function and role of the H3-T6SS. Genetic inactivation of the H3-T6SS disrupts *P. aeruginosa* virulence in murine lung and burn infection models; however, the molecular mechanisms by which H3-T6SS effectors contribute to infection remain unknown [62]. Two effectors of the H3-T6SS have been identified, referred to as TepB and TseF [86,87]. TepB is delivered to mammalian epithelial cells by the H3-T6SS of *P. aeruginosa* strain PA14, but its biochemical activity and physiological consequence on these cells remain to be elucidated [86]. It is important to note that genetic inactivation of either *tepB* or the entire H3-T6SS appears to profoundly impair both twitching motility and biofilm formation, which are known to play important roles in *P. aeruginosa* virulence [86,96]. This finding suggests that the perturbation of the H3-T6SS may have unintended consequences on the physiology of *P. aeruginosa* and, therefore, may confound the conclusion that this system directly contributes to virulence. However, the effect of H3-T6SS activity on motility and biofilm formation may represent an unknown regulatory role for the H3-T6SS substrate accumulation in the cytoplasm. Such a regulatory mechanism has been described in *Vibrio cholerae* and some evidence suggests that Hcp protein accumulation may control the *P. aeruginosa* H2-T6SS [97]. Whether a similar mechanism underlies the control of twitching motility and virulence by the H3-T6SS remains unknown but could present an opportunity to better understand the connection between these *P. aeruginosa* virulence determinants.

The only other protein reported to transit the H3-T6SS is TseF but, like TepB, its biological activity is contentious [87]. Unlike all previously described T6SS effectors, TseF does not function as a cellular toxin but rather is thought to participate in iron uptake. In a genetic background lacking three known iron-acquisition systems (pyoverdin, pyochelin and ferrous iron transport), *tseF* was shown to be required for full growth in iron-depleted media. Furthermore, TseF was found to interact with outer membrane vesicles (OMVs) containing iron (Fe^2+^) bound to the *Pseudomonas* quinolone signal (PQS), an iron-responsive quorum sensing molecule. Interestingly, TseF does not interact with iron directly. TseF was found to recruit these vesicles to the outer membrane transporters FptA and OprF, thus facilitating iron acquisition [87]. While these preliminary findings are intriguing, further study is required to conclusively implicate TseF in metal uptake. The structure of TseF and the molecular basis for its interaction with PQS in OMVs remains unknown. Additionally, the finding that TseF can only interact with iron bound to PQS and not free iron is surprising since all known *P. aeruginosa* siderophores bind to iron directly [98]. Lastly, the purported iron acquisition function of the H3-T6SS differs dramatically from the toxin delivery role of the H1- and H2-T6SSs. It is unclear how this system could function so differently from the H1- and H2-T6SS, especially given the structural similarities of the apparatus components between these systems. Further study of iron acquisition by the H3-T6SS may provide insight into the apparent evolutionary divergence of this system from other characterized T6SSs.

## Conclusions and future directions

Understanding the toxin repertoires and regulatory paradigms of the three T6SSs of *P. aeruginosa* has been a major area of T6SS research since the discovery of this secretion system nearly 20 years ago [99]. The mechanistic study of these systems has focused on biochemically characterizing the repertoire of toxins they export and elucidating the effects of these toxins on target cells. Furthermore, advances in our understanding of the pathways that regulate these three systems offer insight into the physiological roles they play in *P. aeruginosa*. The current collection of evidence implicates the H1-T6SS as a defensive weapon of interbacterial antagonism used to protect *P. aeruginosa* against threats posed by competitor bacteria. By contrast, the H2- and H3-T6SS appear to play an offensive role in *P. aeruginosa* virulence.

While much progress has been made in understanding the T6SSs of *P. aeruginosa*, many questions remain to be answered. The available evidence strongly implicates the H1-T6SS as a dedicated weapon of interbacterial competition. The H2-T6SS, in contrast, is frequently described as a trans-kingdom secretion system that delivers toxins to both eukaryotic and bacterial cells. There is no question that H2-T6SS toxins are delivered to and kill bacteria under laboratory conditions, and the existence of cognate immunity proteins that confer protection against these toxins implies that this activity applies a selective pressure to *P. aeruginosa* in nature. Furthermore, at least one H2-T6SS toxin, VgrG2b, targets a cellular compartment that is unique to bacteria and is, therefore, likely only effective against this cell type, which implicates the H2-T6SS in interbacterial competition [82]. The biological significance of these toxins towards eukaryotic cells, however, remains unclear. It should perhaps be expected that effectors that target universally conserved macromolecules, such as RNA or phospholipids, are toxic to eukaryotic as well as bacterial cells. The finding that these toxins, which appear to indiscriminately kill bacteria, can subtly perturb host cell physiology and promote *P. aeruginosa* virulence is more surprising [92,94]. It remains to be shown how a toxin with a single described biochemical activity can have such different effects in eukaryotic versus prokaryotic cells. The molecular mechanisms of these phenomena and the selective pressures that underlie their evolution, therefore, represent exciting areas for further investigation.

The H3-T6SS also represents uncharted territory for future study. While two effectors that transit this system have been described, their molecular mechanisms remain poorly characterized [86,87]. Understanding the precise mechanisms of these effectors and identifying other effectors exported by the H3-T6SS may provide deeper insight into the role this system plays in *P. aeruginosa* virulence. Additionally, existing evidence suggests that the H3-T6SS gene expression is co-ordinated with that of the H2-T6SS. If and how the H3-T6SS is induced independently of the H2-T6SS remains an open question and may reveal the functions of this system that make it unique from the H2-T6SS.

Overall, the three T6SSs of *P. aeruginosa* appear to play distinct physiological roles in the diverse lifestyles of this organism. By delivering a broad repertoire of functionally diverse toxins into both prokaryotic and eukaryotic cells, these systems contribute to *P. aeruginosa* competition with other bacteria in its environment, overcome the protective host microbiome, and perturb the physiology of host cells during infection. By encoding three distinct systems with their own regulatory controls and toxin arsenals, *P. aeruginosa* can respond to multiple extracellular signals and export toxins that are most likely to be effective in a particular ecological context. Further investigation is required to better understand the environmental cues that control the activity of these systems and to fully appreciate the role of these systems in clinical and environmental isolates of *P. aeruginosa*.

## Abbreviations

CTD: C-terminal domains
Hcp: haemolysin co-regulated protein
OMVs: outer membrane vesicles
PAAR: proline-alanine-alanine-arginine
PQS: Pseudomonas quinolone signal
Rhs: recombination hotspot
TPP: threonine phosphorylation pathway
T6SS: Type VI secretion system
Tap: T6SS adaptor protein
Tse: T6SS effectors
VgrG: valine glycine repeat protein G
